# Transcriptome analysis of the differences in gene expression between testis and ovary in green mud crab (*Scylla paramamosain*)

**DOI:** 10.1186/1471-2164-15-585

**Published:** 2014-07-11

**Authors:** Jie Gao, Xiaowei Wang, Zhihua Zou, Xiwei Jia, Yilei Wang, Ziping Zhang

**Affiliations:** Key Laboratory of Healthy Mariculture in the East China Sea, Ministry of Agriculture, Fisheries College, Jimei University, 361021 Xiamen, China; Department of Natural Sciences and Mathematics, State University of New York at Cobleskill, 12043 Cobleskill, NY USA

**Keywords:** Testis, Ovary, Specifically/differentially expressed genes, Transcriptome, *Scylla paramamosain*

## Abstract

**Background:**

The green mud crab (*Scylla paramamosain*) is the most prevalent crustacean on the southeast coast of China. The molecular regulatory mechanism of sex determination and gonadal differentiation in this species has received considerable attention in recent years because of the huge differences—both biological and economic—between male and female crabs. In this study, next-generation sequencing technology was used to develop deep-coverage transcriptomic sequencing data for the testis and ovary of *S. paramamosain*.

**Results:**

A total of 365,116 reads (testis 171,962, ovary 193,154) with an average sequence length of 285 bp were produced from testis and ovary cDNA libraries. After filtering out contaminating reads, the clean reads were assembled, producing a total of 21,791 isotigs and leaving 22,814 reads as singlets. Using the BLASTX program, 3,471 unique sequences (2,275 isotigs and 1,196 singletons) were annotated with known protein sequences from the NCBI non-redundant (Nr) protein sequence database. The Gene Ontology and KEGG (Kyoto Encyclopedia of Genes and Genomes) analyses allowed the 224 unique sequences that were annotated with enzyme code (EC) numbers to be mapped into 174 KEGG pathways. After comparing the ovary and testis libraries, 4,021 gonad-differentially, 10,522 ovary-specifically, and 19,013 testis-specifically expressed genes were identified. Moreover, 33 ovary-specific, 14 testis-specific, and 34 gonad-differential transcripts were confirmed by semi-quantitative PCR and quantitative real-time PCR. In addition, 8,610 putative simple sequence repeats (SSRs) and 23,879 potential single nucleotide polymorphisms (SNPs) were identified.

**Conclusion:**

This is the first large-scale RNA sequencing of *S. paramamosain* to be reported. We have identified many important functional genes and made a preliminary attempt to construct the regulatory network involved in the gonadal development of crustaceans. The annotated transcriptome data will provide fundamental support for future research into the reproduction biology of *S. paramamosain*. A large number of candidate SSRs and SNPs were detected, which could be used as genetic markers for population genetics and functional genomics in this species.

**Electronic supplementary material:**

The online version of this article (doi:10.1186/1471-2164-15-585) contains supplementary material, which is available to authorized users.

## Background

Many male and female crabs have a number of significant differences in their biology and economic value, such as growth rate and body size. A study of gonadal differentiation and development in crabs would be helpful for the aquaculture industry. Among the four species of the genus Scylla, the green mud crab (*Scylla paramamosain*) is the most prevalent species in the southeast coast of China [1–3], and it has become one of the most important marine aquaculture species in China because of its huge economic value. At present, however, the demand for this crab far exceeds the supply. A major challenge currently facing the crab aquaculture industry is the need to form a series of viable seed production techniques to help increase supply. Accordingly, it is important to understand the regulatory mechanisms of sex determination, gonadal differentiation and maturation, and gametogenesis in this species. To understand these mechanisms at the molecular level in more detail, the regulatory pathways associated with gonadal differences need to be studied at the genomic level.

Many methods, such as traditional suppression subtractive hybridization [4–8], annealing control primer [9–11], differential display RT-PCR (DDRT-PCR) [12, 13], EST sequencing [14, 15], and microarray [15], have been applied in research into the crustacean reproductive system, and many differentially expressed genes involved in sex determination, gametogenesis, and gonadal differentiation and maturation have been identified and characterized. Some of these genes were detected in crabs; for example, cell division cycle 2 (*cdc*2) [16], *cyclin B* [16], extracellular regulated protein kinase 2 (*Erk*2) [17], and small ubiquitin-related modifier 1 (*SUMO*-1) [18] in *S. paramamosain*, and double-sex and mab-3 related transcription factor (*Dmrt*) [19] in *Eriocheir sinensis*. Several of the differentially expressed genes were found in shrimps; for example, *Tra*-2 [4], mitogen-activated protein kinase 1 (*Mapk*1) [20], prostaglandin reductase 1 [21], and ubiquitin specific peptidase 9, X-linked (*USP9X*)[22] in *Penaeus monodon*, and proliferating cell nuclear antigen (*PCNA*) [11], ubiquitin-conjugating enzyme E2r (*UBE2r*) [10], and heat shock protein 90 (*HSP*90) [23] in *Marsupenaeus japonicus*. However, because of the limited amount of genomic data that is available for crustaceans, it is difficult to identify genes and construct regulatory networks associated with crab reproduction.

Expressed sequence tags (ESTs) represent effective transcription fragments of whole genomes, avoiding non-coding and repetitive sequences. ESTs contain a huge amount of functional information that can greatly accelerate the research and discovery of new genes [24] and molecular markers [25, 26]. Next-generation high-throughput sequencing technologies have been used to generate large amounts of large-scale transcript sequences and gene expression data for many species when the genomic sequences are not available. These technologies include the Roche/454 platform [27], the Illumina/Solexa system [28], and the ABI SOLiD technology [29], all of which have high-accuracy, high-speed, and low-cost compared with first-generation sequencing technology (Sanger sequencing).

Here a Roche/454 GS FLX sequencing system was used for deep-coverage RNA sequencing of *S. paramamosain* during different gonadal developing stages to generate a transcriptome database that will enlarge the public EST database for this species and help support future studies. The transcriptomic analysis will help to identify gonad-specific/differential genes by comparing the transcripts in the testis and ovary libraries, which will help build a more complete understanding of the regulatory mechanisms associated with gonadal differentiation and reproductive processes.

## Results

### 454 sequencing and assembly of green mud crab cDNA libraries

**Figure 1:**
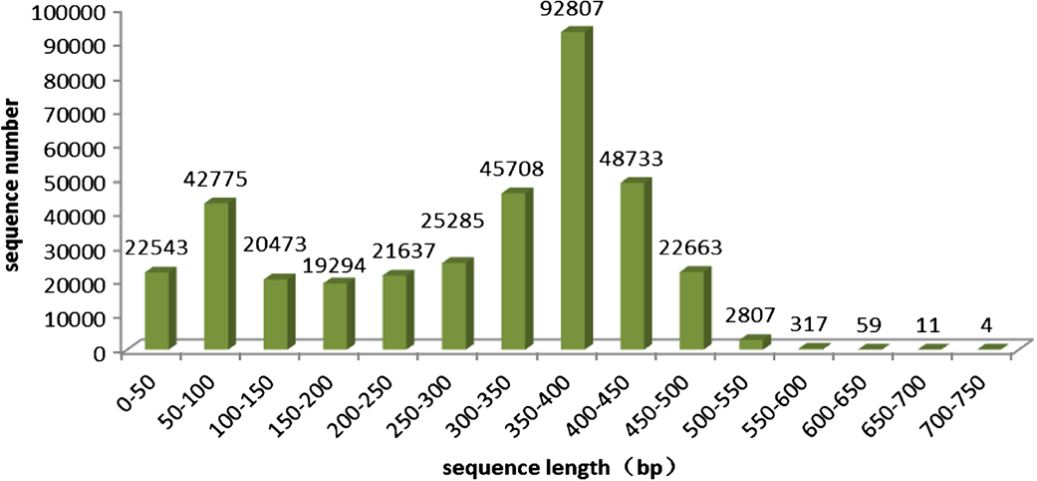
**Length distribution of all reads generated by 454 sequencing.** X-axis:Size distribution of all raw reads,Y-axis:The number of reads in different length range. Of all the reads, more than half (187,248, 51.3%) of them were between 300 bp and 450 bp. The longest read was 735 bp and the shortest read was less than 50 bp.

Two cDNA libraries were constructed from the testes and ovaries of green mud crabs. The Roche/454 sequencing generated 171,962 reads from the testis and 193,154 reads from the ovary with average sequence lengths of 290 bp and 281 bp for the testis and ovary reads respectively. More than half the reads (187,248, 51.3%) were between 300 bp and 450 bp in length, the longest read was 735 bp and the shortest read was less than 50 bp, and the overall length of the reads was 104 Mb. The size distribution of the raw reads is shown in Figure 1. The raw reads from these two libraries have been deposited in GenBank with the following Accession Numbers: [GenBank:SRA074281] and [GenBank:SRA074282].

**Figure 2:**
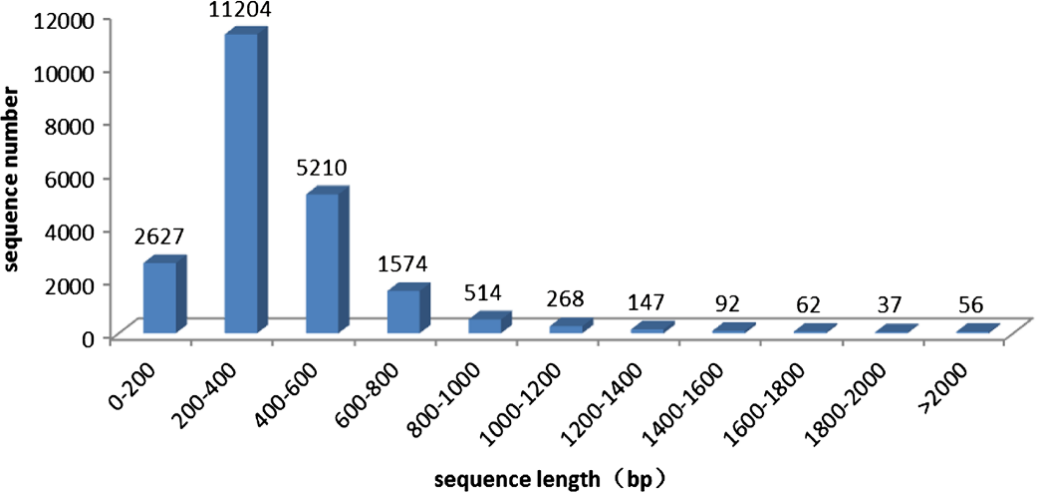
**Length distribution of the assembled isotigs.** X-axis:Size distribution of assembled isotigs,Y-axis:The number of isotigs in different length range. The length of isotigs varied from 100 to 2000 bp, more than three fourths (16414, 75.3%) of the isotigs occupied a range of 200-600 bp long.

After filtering out the adaptor primers and low-quality and very short (<50 bp) reads, 298,605 (81.8%) clean reads with an average sequence length of 274 bp remained. The clean reads in the two libraries were assembled separately. Altogether, 21,791 isotigs (testis 12,439, ovary 9,352) were assembled, leaving 22,814 reads (testis 14,241, ovary 8,573) as singlets. The isotigs varied from 100 to 2,000 bp in length, and the singlets varied from 50 to 500 bp in length. More than three-fourths of the isotigs (16,414, 75.3%) were from 200 to 600 bp long. A statistical analysis of the assembly is presented in Table 1, and the length distribution of the isotigs is shown in Figure 2.

**Table 1:** **Summary statistics of the RNA sequencing of the** ***S.paramamosain***

Reads	Testis	Ovary	Total
Total number of raw reads	171962	193154	365116
Average length of raw reads(bp)	290	281	285
overall length of the reads(Mb)	50	54	104.00
total number of trimmed reads	138967	159638	298065
Total number of isotigs	12439	9352	21791
Total number of singlets	14241	8573	22814
**The comparison between two libraries,**			
Common genes			6395
Differentially expressed genes with significant difference (P <0.05)	\	\	4199
Ovary-specifically expressed genes	\	10522	\
Testis-specifically expressed genes	19013	\	\

### Annotation of the unique sequences in the two libraries

**Figure 3:**
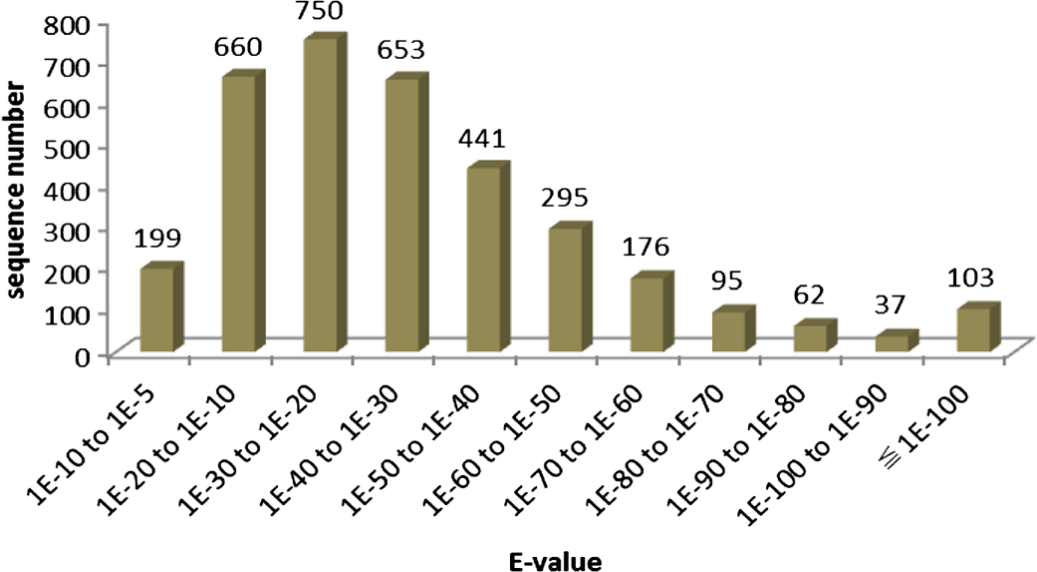
**Distribution of the E-values for annotated sequences.** X-axis:E-values distribution of annotated sequences,Y-axis:The number of sequences in different E-values range. In total, 3,272 (94.3%) unique sequences share significant similarity with the matched sequences (E≤1e-10), while 199 (5.7%) of unique sequences left share weak similarity with the matched sequences with E value between 1e-10 to 1e-5.

Annotation of the assembled (unique) sequences was carried out using BLASTX searches against the NCBI non-redundant (Nr) protein sequence database. We identified 3,471 unique sequences (testis 1,718, ovary 1,753) that shared similarities with known protein sequences; 3,272 (94.3%) sequences shared significant similarities with the matched sequences (E ≤1e-10), while the other 199 (5.7%) sequences shared a weak similarity (E 1e-10 to 1e-5). The E-value distribution for the annotated sequences is shown in Figure 3.

**Figure 4:**
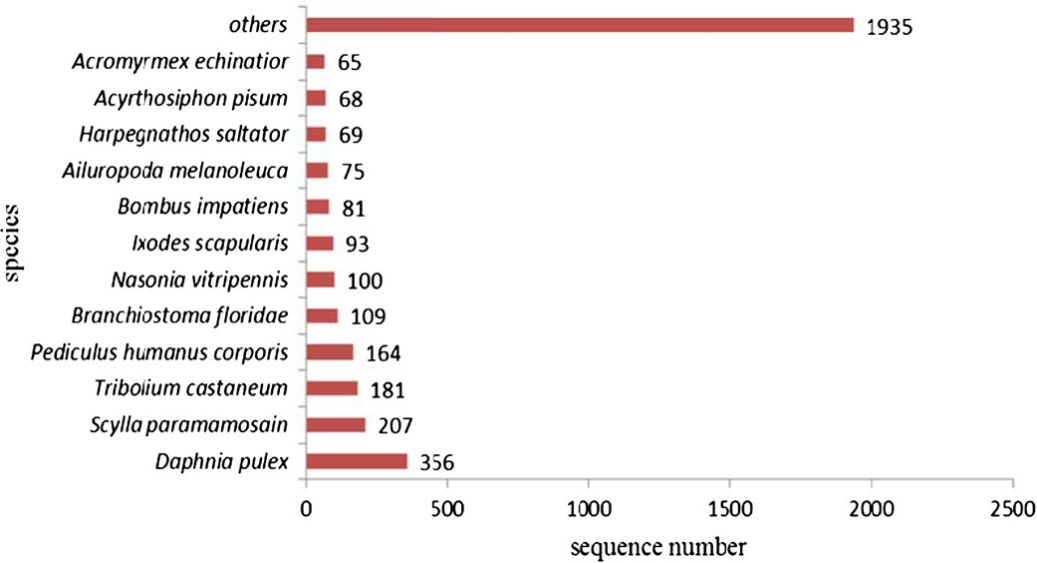
**Species that match to the annotated sequences of** ***S. paramamosain.*** X-axis:The number of annotated sequences matching each species, Y-axis:Distribution of top species that match to the annotated sequences.

The 3,471 annotated sequences matched known sequences from 362 different species. Most numbers of sequence hits were to the water flea *Daphnia pulex* (10.16%), the red flour beetle *Tribolium castaneum* (5.17%), the body louse *Pediculus humanus corporis* (4.68%), and the Florida lancelet *Branchiostoma floridae* (3.11%). Except for the top 12 species with sequence hits, 1,935 (55.24%) of the annotated sequences matched sequences from other species (Figure 4). The top six crustacean species with the most number of sequence hits were *S. paramamosain* (207, 5.92%), *P. monodon* (44, 1.26%), *Litopenaeus vannamei* (43, 1.23%), *E. sinensis* (37, 1.06%), *Procambarus clarkia* (30, 0.86%), and *Homarus americanus* (28, 0.80%).

### Gene ontology analysis

**Figure 5:**
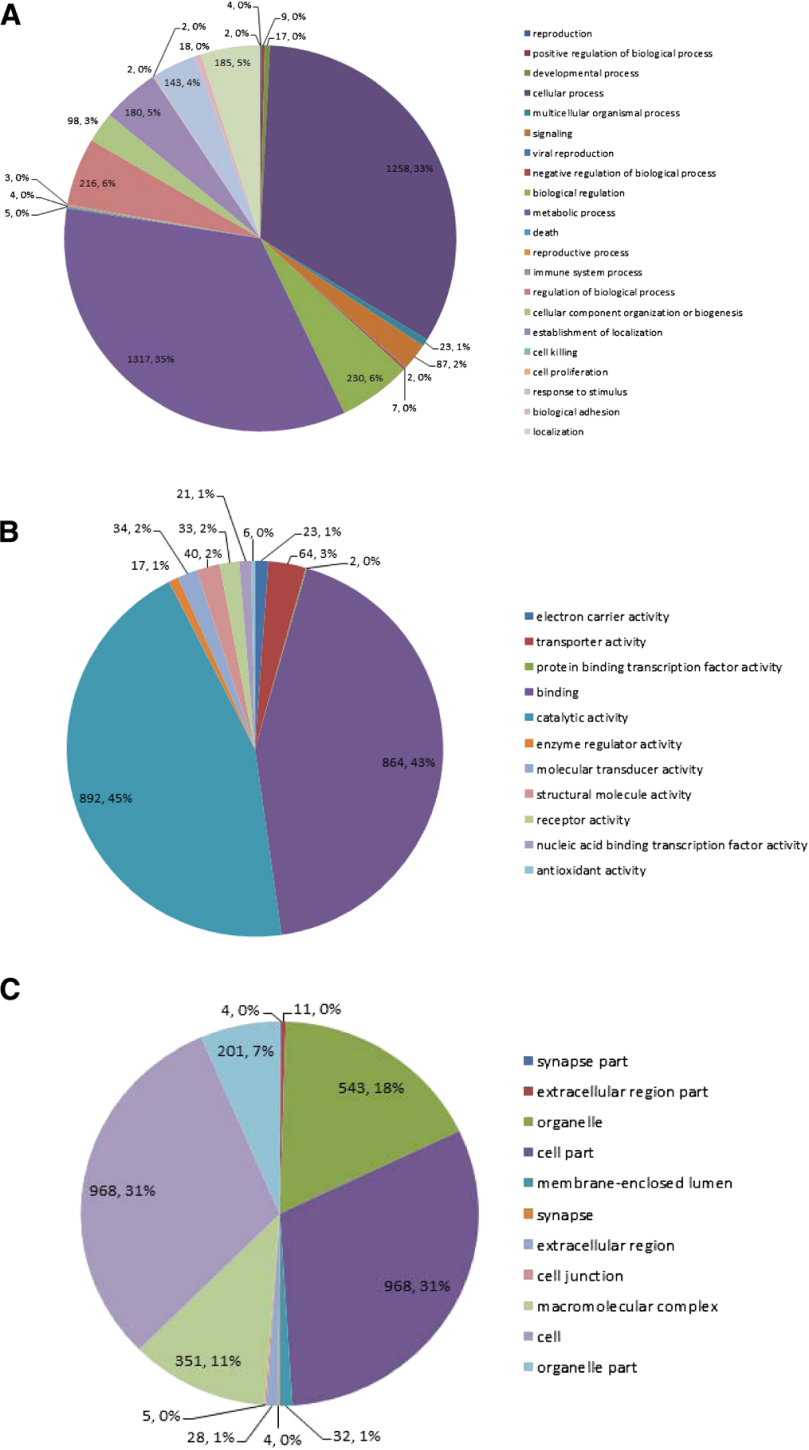
**Distributions of the annotated sequences in three GO categories (Level Two). A**: biological process, **B**: molecular function, **C**: cellular component.

The Blast2GO program was used to assign Gene Ontology (GO) terms to the 3,471 annotated sequences to predict the functions of the unique sequences and the translated proteins that they encode. A total of 1,540, 995, and 1,368 sequences were assigned to the biological process, cellular component, and molecular function GO categories, respectively. The distributions of the annotated sequences in the three GO categories (level 2 terms) are shown in Figure 5. In the biological process category, the metabolic process (34.55%) and cellular process (33.00%) level 2 terms were the most abundant terms. In the molecular function category, catalytic activity (44.69%) and binding (43.29%) were the most abundant, while, in the cellular component category, cell (31.08%), cell part (31.08%), organelle part (17.43%), and macromolecular complex (11.27%) were the most abundant level 2 terms.

### Functional classification based on KEGG analysis

**Figure 6:**
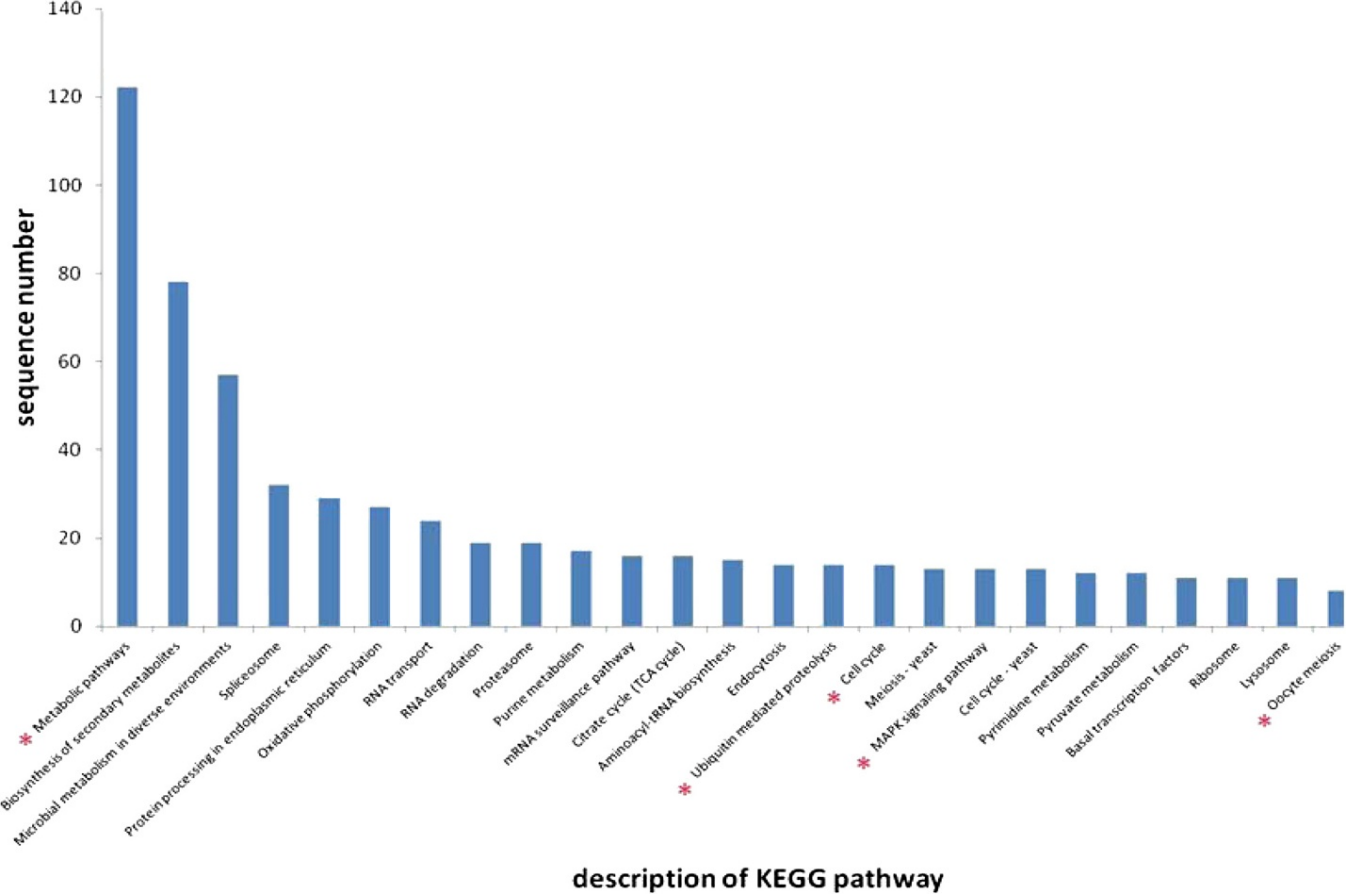
**Distribution of the mapped KEGG pathways.** X-axis: distribution of KEGG pathways, Y-axis: the number of sequences mapped into each KEGG pathway *represents important pathways.

Functional classification and pathway assignments were based on a Kyoto Encyclopedia of Genes and Genomes (KEGG) analysis. Among the 3,471 annotated sequences, 224 that were annotated with an enzyme code EC number were mapped to 174 KEGG pathways. The top five KEGG pathways were metabolic pathways, biosynthesis of secondary metabolites, spliceosome, microbial metabolism in diverse environments, and oxidative phosphorylation pathway. Some sequences were mapped to several pathways related with reproduction, growth, development, and immunity; for example, cell cycle, MAPK signaling pathway, ubiquitin mediated proteolysis, oocyte meiosis, progesterone-mediated oocyte maturation, GnRH signaling pathway, Wnt signaling pathway, Hedgehog signaling pathway, p53 signaling pathway, and Toll-like receptor signaling pathway. The distribution of the mapped KEGG pathways is presented in Figure 6.

### Analysis of gonad-differentially and specifically expressed genes

**Figure 7:**
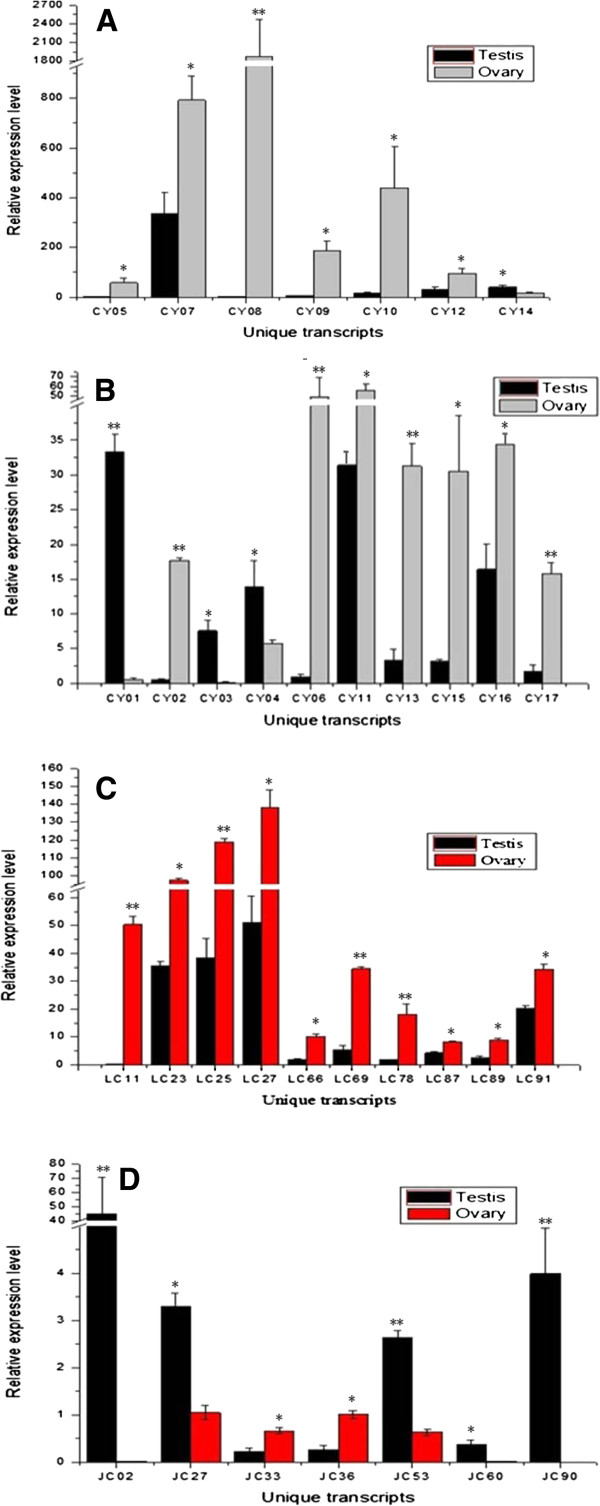
**Expression analysis of gonad differentially expressed transcripts by qRT-PCR.** X-axis: The serial number of gonad differentially-expressed transcripts, Y-axis: Relative expression of each transcripts. 18S rRNA is as internal control gene. *indicates significantly differential expression(*P* < 0.05), **indicates most significantly differential expression (*P* < 0.01). **A**&**B** 60 differentially expressed transcripts were chosen for quantitative real-time PCR (qRT-PCR), qRT-PCR result showed that 17 (4 testis-predominant, 13 ovary-predominant) were confirmed as gonad-differentially expressed. **C** 68 ovary-specifically expressed transcripts were chosen for semi-quantitative PCR validation firstly. Then, 35 transcripts that expressed both in ovary and testis were performed for qRT-PCR. Result showed that 10 transcripts were expressed significantly higher in ovaries than in testes. **D** 55 testis-specifically expressed transcripts were chosen for semi-quantitative PCR validation firstly. Then, 41transcripts that expressed both in ovary and testis were performed for qRT-PCR. Result showed that 7 transcripts (5 testis-predominant, 2 ovary-predominant) were expressed in ovaries and testes with significant difference.

**Figure 8:**
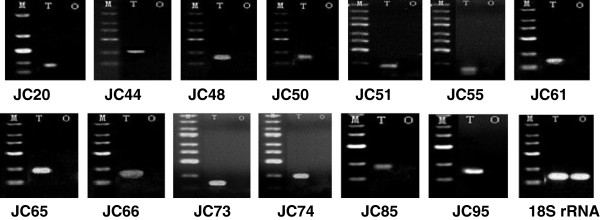
**Expression analysis of testis-specifically expressed transcripts by semi-quantitative PCR.** JC represents the serial number of testis-specifically expressed transcripts, 18S rRNA represents endogenous control. M: marker, T: testis, O: ovary.

**Figure 9:**
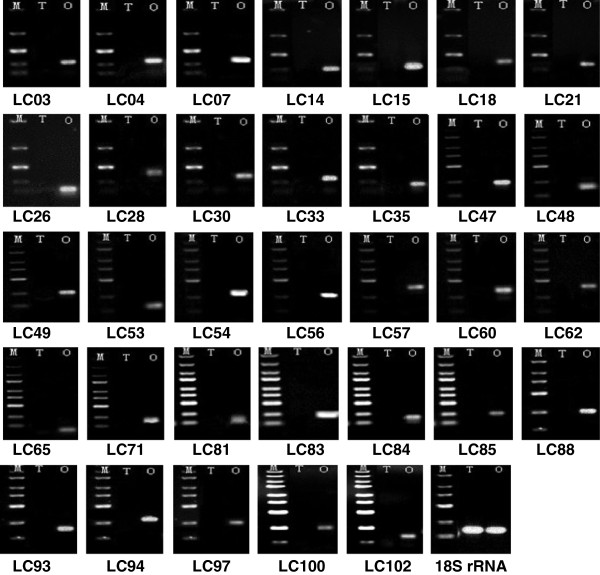
**Expression analysis of ovary-specifically expressed transcripts by semi-quantitative PCR.** LC represents the serial number of ovary-specifically expressed transcripts, 18S rRNA represents endogenous control. M: marker, T: testis, O: ovary.

By comparing the sequences in the ovary and testis libraries, 4,021 gonad-differentially expressed genes (Fisher *P* test, adjusted using the false discovery rate), 10,522 ovary-specifically expressed genes and 19,013 testis-specifically expressed genes were identified. From these, 60 differentially, 55 testis-specifically, and 68 ovary-specifically expressed transcripts were chosen for validation by quantitative real-time PCR (qRT-PCR) and semi-quantitative PCR. As shown in Figure 7A and B, the qRT-PCR result confirmed that 17 (four testis-predominant and 13 ovary-predominant) of the 60 differentially expressed transcripts were gonad-differentially expressed. This finding corresponds with the results from the Fisher *P* test. Semi-quantitative PCR confirmed that 33 of the 68 ovary-specifically expressed transcripts were expressed only in the ovaries (Figure 8), while the other 35 transcripts were expressed in both ovaries and testes. Moreover, among these 35 transcripts, ten were found to be expressed significantly higher in ovaries than in testes by qRT-PCR validation (Figure 7C). Similarly, only 14 of the 55 testis-specifically expressed transcripts were verified as testis-specifically expressed by semi-quantitative PCR (Figure 9). The expressions of the other 41 transcripts were examined by qRT-PCR, and the result showed that only seven of them (five testis-predominant and two ovary-predominant) were significantly differentially expressed in ovaries and testes (Figure 7D).

### Discovery of molecular markers

Isotigs and singlets were both used to predict simple sequence repeats (SSRs), and isotigs alone were used to predict single nucleotide polymorphisms (SNPs). Based on the number of reads (represented by “n”) that aligned to a SSR or SNP, the number of SSRs and SNPs were calculated when n ≥1, n ≥3, and n ≥5 (Additional file 1: Tables S1 and S2). We found that when the n value was increased, the numbers of predicted SSRs and SNPs decreased significantly but the accuracy was greatly improved.

**Figure 10:**
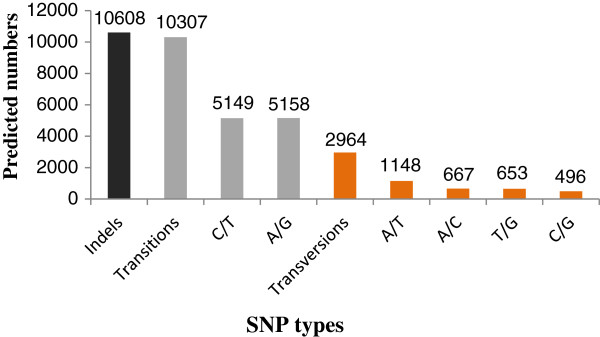
**Distribution of putative single nucleotide polymorphisms (SNP) in the transcriptome of** ***S. paramamosain.*** X-axis: Distribution of SNP types, Y-axis: The number of different SNP types.

For n ≥1, we obtained 8,610 SSRs from the ovary and testis transcriptome libraries (Table 2). The largest number of SSR motifs were dinucleotides (5,265), which accounted for 61.43% of all the predicted SSRs, followed by trinucleotides (2,717, 31.70%), tetranucleotides (479, 5.59%), and penta/hexanucleotides (149, 1.28%). In addition, of the 6,707 SSR-containing sequences, 1,427 sequences were predicted to contain more than two of the SSR types.Using the ssahaSNP software, 10,608 indels and 13,271 putative SNPs were detected from 5,295 isotigs (n ≥1). The predicted SNPs included 10,307 transitions and 2,964 transversions. Indels were the most common SNP type, making up 44.42% of the SNP types, while transitions (43.16%) and transversions (12.42%) made up the rest. The most frequent SNPs types were A/G, C/T, and A/T, while C/G was the least common type (Figure 10).

**Table 2:** **Summary of simple sequence repeat (SSR) in the transcriptome of** ***S.paramamosain***

SSR type	Number of SSRs in ovary pool	Number of SSRs in testis pool	Total number	Percentage of total
Di-nucleotide	2679	2586	5265	61.43%
Tri-nucleotide	1300	1417	2717	31.70%
Tetra-nucleotide	218	261	479	5.59%
Penta-nucleotide	40	49	89	1.04%
Hexa-nucleotide	2	19	21	0.24%
total	4239	4332	8571	100.00%

## Discussion

Large-scale RNA sequencing of the green mud crab *S. paramamosain* (testes and ovaries during different gonadal developing periods) was performed to expand the limited amount of sequence data that are available for crabs in the public EST database. Our aim was to identify potentially valuable novel genes that can be used to attain a better understanding of the basic biological mechanisms of reproduction, development, and immunity in crabs.

The RNA sequencing produced 365,116 reads, of which 298,605 were high-quality reads, which were then assembled into 44,605 unique sequences. Only 3,471 (7.78%) of the unique sequences were annotated successfully by BLASTX searches against the Nr database, probably because of the limited number of crustacean sequences in public databases. Besides, some of the unannotated sequences may be 3’ or 5’ untranslated regions, non-coding RNAs, or short sequences that do not contain known protein domains. Because sequence similarity is not necessarily indicative of functional homology, in future studies, the functions of these annotated sequences will need to be validated. Further, the model species *D. pulex* was the first crustacean to have its whole genome sequenced [30], therefore, it is not surprising that the *S. paramamosain* sequences had the highest number of matches with *D. pulex* sequences*.* Even then, only about 10.16% of all the annotated sequences matched *D. pulex*, probably because *S. paramamosain* belongs to the class Malacostraca while *D. pulex* belongs to the class Branchiopoda*.*

GO and KEGG analysis provide gene function classification labels and gene function background knowledge that can help predict the role of protein interaction networks in cells [31, 32]. In the GO analysis, the most abundant level 3 GO terms in the biological process category, were cellular metabolic process, primary metabolic process, macromolecule metabolic process, nitrogen compound metabolic process, and biosynthetic process. A similar approach has been applied successfully in the transcriptomic analysis of the housefly *Musca domestica* larva [33] and platy fish *Xiphophorus maculatus* [34].

The focus of our project was to mine gonad-differentially/specifically expressed genes. We identified 6,395 common genes in the testis and ovary libraries (the method is described in “Materials and Methods”), of which 4,021 (65.66%) were considered to have a significant difference (Fisher *P* test*, P* <0.05), and 639 (15.22%) of them were annotated successfully. The remaining 84.78% of the common genes could not be annotated. Besides, 10,522 ovary-specifically expressed genes (7.18% annotated, 92.82% unannotated) were identified in the ovary library and 19,013 testis-specifically expressed genes (4.43% annotated, 95.57% unannotated) were identified in the testis library. Three issues arise from these findings. First, the number of unannotated sequences was much larger than the annotated ones, which made it is almost impossible to exploit the unannotated sequences, specifically the differentially/specifically expressed genes, meaning that potentially useful genetic information was missed. Second, the number of testis-specific transcripts was much more than the number of ovary-specific transcript, indicating that female original development was in a kind of “default” state. It has been reported that the initial male development occurred by activating a series of testis-specific genes directly or indirectly and/or by repressing genes that were specifically involved in ovarian development [34, 35]. Third, the annotation ratio of the testis sequences was less than that of the ovary sequences. This could be because the study of female animals has been the key focus of aquaculture and researchers have paid much more attention to female crustaceans than to male crustaceans. Correspondingly, genetic information for the female of species is much richer than for the male.

Among all the 60 tested gonad-differentially expressed transcripts, four testis-predominant transcripts, and 13 ovary-predominant transcripts were confirmed by qRT-PCR (Figure 7A and B). In addition, 33 ovary-specifically expressed transcripts and 14 testis-specifically expressed transcripts were verified. In addition, another 17 specifically expressed transcripts were proved to be significantly differentially expressed in ovaries and testes (Figure 7C and D). We speculate that all these transcripts could play important roles in certain parts of the gonadal differentiation physiological process. We failed to confirm several differentially/specifically expressed transcripts probably because the RNA samples came from different sources: the RNA used for Roche/454 sequencing came from testes/ovaries pools at various developing stages, while the RNA for the semi-quantitative PCR and qRT-PCR verification were from the mature stage of the gonads (see “Sample preparation and RNA extraction” section for details). Perhaps the unconfirmed transcripts were expressed in immature stages rather than in the mature stage of gonad development. Besides, some differences often exist between the bioinformatics analysis of next-generation sequencing technology and the actual experimental analysis. Interestingly, these findings indicate that there might be many more valuable genes to mine and research in this large-scale RNA sequencing dataset.

Despite the various limitations described above, a large number of key genes and some important pathways that may participate in the gonadal development and reproductive process were detected in the transcriptomic sequencing data (Additional file 1: Table S3). They include the cyclin-CDK-CKI pathway, ubiquitin protease pathway, sumoylation (SUMO) pathway, MAPK signal transduction pathway, and the antioxidant system.

We identified the *vasa* gene [GenBank:GU187045] in our transcriptome database. *Vasa* is specifically expressed in gonads and plays an important role in germ cell formation and gametogenesis in *S. paramamosain* [36]. We also discovered some genes such as *Dmrt* and Feminization-1 (*FEM-1*) that have not been identified previously in *S. paramamosain. Dmrt* has been reported to be involved the regulation of sex determination/differentiation pathway in most vertebrates and invertebrates [19, 37, 38] and *FEM-1* was reported to be required for all aspects of testis development [39, 40]. By qRT-PCR validation, we found that *Dmrt* was specifically expressed in the testis, as reported previously in *E. sinensis* [19], while *FEM-1* was expressed similarly in both ovaries and testes (Additional file 1: Table S3). Whether *FEM-1* participates in the process of sex differentiation or gonad development in the crab has yet to be established.

Vitellogenesis, the process of yolk formation and accumulation, is central to oogenesis and ovarian maturation. In oviparous vertebrates, estrogen hormones, especially the 17β-estradiol E2, increase the synthesis of vitellogenin (Vtg) through the enhancement of the transcription of the *Vtg* gene. This process is mediated by an estrogen receptor (ER) and heat shock protein 90 (Hsp90) [41, 42]. Yano and Hoshino showed that E2 can stimulate synthesis of vitellogenin and oocyte development in *M. japonicas* [43] *.* A study of *Metapenaeus ensis* also indicated that Hsp90 had some role in the regulation of vitellogenin synthesis [42]. Moreover, the discovery of ER in mollusks and the estrogen receptor-related receptor (ERR) in *Neomysis japonica* suggested that a similar mechanism in vertebrates also exists in crustaceans. Although an ER has not been found yet, we identified *Vtg*, vitellogenin receptor (*VgR*), *HSP90*, and *ERR* in our transcriptomic data. *Vtg* [GenBank:FJ8120900] was previously found to be specifically expressed in the ovaries and hepatopancreas [44]. In the present study, *VgR* was specifically expressed in ovaries (Additional file 2: Figure S1), *ERR* were expressed similarly in both ovaries and testes (Additional file 1: Table S3), while *HSP90* exhibited a higher expression level in ovaries than in testes (*P* <0.5) (Additional file 1: Table S3). More work is needed to confirm whether ERR has the same function of ER and whether the E2-ER-Hsp90-Vtg pathway exists in crustaceans.

Besides the ER-mediated classic nuclear receptor pathway, estrogen can also bind with its corresponding membranous receptors to start a fast non-genetic effect. The G protein-coupled receptor 30 (GPR30) has been regarded as a new type of estrogen receptor [45]. Wang et al. [46] and Sirianni et al. [47] found that *GPR30* mRNA was expressed in ovarian theca cells and in the granular cells of hamster and the mouse spermatogonial cell line. This finding indicated that GPR30 played a role in the reproductive system. The complex of estrogen (E2) and GPR30 have been reported to activate the ERK pathway and accelerate the process of cell cycle, so as to promote cell growth and proliferation [48]. Some researchers believe that there is a relationship between GPR30 and the classic nuclear receptor ER. We found the G protein-coupled receptor 89 (*Sp-gpr*89) and G-coupled receptor kinase 2 in the transcriptome sequencing data. The qRT-PCR result showed that *Sp-gpr*89 [GenBank:HM036654.1] and G-coupled receptor kinase 2 were expressed in both the mature ovaries and testes with no differences (Additional file 1: Table S3). Further, our results (Additional file 2: Figure S2) showed that the expression level of *Sp-gpr*89 in the ovaries was significantly higher (*P* <0.05) compared with other tissues (except testes). As the ovary develop, *Sp-gpr*89 expression levels rose gradually, and the expression level in the mature ovary (O5) was significantly different than the expression levels in the other four stages (*P* <0.05). Further investigations are needed to determine whether the function of GPR89 in crab is similar to the function of GPR30.

Oocyte maturation is a complex process that is regulated by multiple factors [49] among which, mature promotion factor (MPF) and mitogen-activated protein kinase (MAPK) play key roles in the process. MPF, a kind of serine/threonine-protein kinase, consists of cyclin B and cell cycle dependent protein kinase 1 (CDK1). When the oocyte cell cycle transforms from the G2 to the M phase, MPF can be activated and this then induces the rapid maturation of oocytes [50]. The ERK1/2 of MAPK family participates mainly in the regulation of oocyte meiosis and is not activated in the immature oocytes. However, ERK1/2 was found to be activated in mature oocytes where it mediated oocytes maturation signals from cytoplasm to nuclei [51]. Oocyte maturation has been a research focus in reproductive biology, but the molecular regulatory mechanism has not been fully elucidated. We found some genes that may be related to this regulatory system in our transcriptomic database, namely, anaphase promoting complex subunit 1, protein phosphatase 2A regulatory subunit A, adenylate cyclase type, and serine/threonine-protein phosphatase PP-V. The qRT-PCR validation results for these genes are shown in Table S3.

The wnt signaling pathway is a conservative signaling network, which takes part in embryonic development, cell differentiation and proliferation, and the process of growth regulation. Wnt is a secreted glycoprotein, and many different *wnt* gene subtypes have been found in a variety of animals. *Wnt*4 is regarded as a sex determination gene, which plays a key role in the morphological development of female mammals [52]. Wnt4 can regulate the formation of the mullerian duct and the generation of ovarian steroids [53]. In female mice that have lost the *wnt4* gene, the mullerian duct did not form, indicating that wnt4 is essential to regulate the reproductive cells in the development of female mice. In male mice, the wnt4 signal system was inactive [54]. Some genes in the wnt signaling pathway, such as wnt6 protein (another member of the wnt family), casein kinase II alpha 1, and protein phosphatase 2A regulatory subunit A were found in our transcriptomic database. *Wnt*6 and protein phosphatase 2A regulatory subunit A were more highly expressed in ovaries than in testes as indicated by the qRT-PCR validation results (Additional file 1: Table S3). Further research is required to elucidate the roles of these genes in the reproductive process of crab.

Vertebrate-type steroids, such as progesterone and 17β-estradiol have been identified in crustaceans. Progesterone stimulates yolk protein synthesis and ovarian maturation in *Penaeus vannamei* [55] and *S. paramamosain* [56], and stimulates pawning in *M. ensis* [57]. The important function of progesterone is mediated by the progestin membrane receptor component (PGMRC). PGRMC1 contains a cytochrome b5 domain fold and belongs to the membrane-associated progesterone receptor (MAPR) protein family that is widespread in eukaryotes [58]. In our study, the expression of *Pgmrc1* in ovaries was significantly higher than in testes (Additional file 1: Table S3). This indicated that *Pgmrc1* may be involved in oogenesis and ovarian development, similar to its involvement in *P. monodon* [59].

OTUB (OTU domain-ubiquitin aldehyde binding protein) belongs to the OTU (ovarian tumor) superfamily of cysteine proteins [60]. There are two subtypes of OTUB in mammals, OTUB1 and OTUB2, which have relative molecular masses of 31 kDa and 27 kDa respectively. OTUB1 has been identified as a novel ERα-interacting protein with the capability of deubiquitinating ERα and then repressing its transcriptional activity [61], which may indicate that OTUB1 plays a major role in the estrogen receptor (ER) signaling pathway in mammals. Our qRT-PCR result showed that the expression levels of *Sp-OTUB* [GenBank:JX195165] in mature ovaries and testes were similar (Additional file 1: Table S3). However, our results published previously indicated that in the process of ovarian development, *Sp-OTUB* was expressed at the highest level in the proliferation stage and exhibited significant differences with its expression in other development stages. In the secondary spermatocytes stage of testis development, the expression of *Sp-OTUB* reached its highest level and was significantly higher than in the primary spermatocytes and spermatids stages [62]. These preliminary results showed that Sp-OTUB may play an important role in reproductive regulation. Its physiological function, however, needs further investigation.

*Wds* (will die slowly, or WD seven) was first identified and characterized in *Drosophila melanogaster* [63, 64]. It is an essential gene that codes for a WD-repeat protein with seven repeats. Northern blots and *in situ* hybridization showed that the expression level of *wds* increased significantly during oogenesis; however, in male germ cells, wds transcript levels were low [63]. Loss of wds function during oogenesis was found to be lethal in *D. melanogaster* [64]. Similarly, the *wds* gene isolated from the Chinese oak silkworm *Antheraea pernyi*, was more abundant in the ovaries than in spermaries [65]. In our study, the qRT-PCR results demonstrated that the expression level of *wds* [GenBank:KC762942] was significantly higher in ovaries than in testes (Additional file 1: Table S3).

SSRs and SNPs are important molecular markers that have been used as powerful tools for genetic mapping, gender identification, population genetic analysis, and breeding. For instance, Li et al. [66] found 33 EST clusters that were involved in immune and defense functions in *Meretrix meretrix.* An ATP-dependent DNA helicase gene, *RuvB-like 2,* with different SNPs in the untranslated region was reported to influence the body weight of *P. monodon* during ovarian development [67]. In addition, two SNPs that were detected within the coding region of the *CYP17-I* gene, were found to be significantly associated with the reproductive traits of *Paralichthys olivaceus* [68]. In this study, a large number SSRs and SNPs were detected from the Roche/454 sequencing data. It is expected that these SSRs and SNPs will play a tremendous role in the exploration and utilization of new genes *S. paramamosain*, and will be developed as a tool for genomics and functional genomics in the future.

## Conclusion

This is the first large-scale RNA sequencing of the green mud crab *S. paramamosain* to be reported. More than 300,000 reads were obtained, and a large number of novel genes were found. Some gonad-differentially and gonad-specifically expressed genes were identified. Many potential SSRs and SNPs were detected that could be used for further genetic breeding, genomic mapping, and gene localization studies. We constructed a deep-coverage EST database from the sequencing data. The read sequences have been deposited in GenBank, thereby enriching the genomic information of crustaceans that is available. Work is still needed to improve the molecular database for understanding the mechanisms of sex determination/differentiation and gonadal differentiation/development in crustaceans. The EST information gathered will contribute greatly to our further studies.

## Methods

### Ethics statement

All of the study design and animal experiments were conducted in accordance with guidelines of Jimei University's Animal Care and Use Committee.

### Sample preparation and RNA extraction

Male and female crabs at different gonadal developing stages (male 55–256g and female 65–283g) were purchased from a commercial farmer in Zhangpu, Fujian, China. Based on the external morphology, color, gonadosomatic index (GSI), and histological features [18], ovarian development was classified into five stages: proliferation (stage I), pre-vitellogenesis (stage II), primary vitellogenesis (stage III), secondary vitellogenesis (stage IV), and tertiary vitellogenesis (stage V). The male crabs were grouped into three stages based on testis development: primary spermatocytes (stage I), secondary spermatocytes (stage II), and spermatids (stage III).

The crabs were placed in an ice bath for 3–5 min until they were lightly anesthetized. Testes and ovaries were then removed quickly, frozen immediately in liquid nitrogen, and stored at –80°C until use. Equal amounts (about 0.5 g) of the ovaries and testes at each developmental stage (six samples each for stage) were mixed and total RNA was extracted using Trizol for the 454 sequencing. Gonad samples of the mature stage (testis stage III, and ovary stage V) were used for the qRT-PCR and semi-quantitative PCR experiments. For most genes, we analyzed only their expression in the mature stage testis and ovary; however, for genes of particular interest, we analyzed their expression in ovaries and testes at each developmental stage.

The integrity of the total RNA was detected by gel electrophoresis and its concentration was determined by measuring the absorbance at 260 nm in a spectrophotometer. cDNA synthesis using a Clonetech SMART kit (Clontech, Palo Alto, CA, USA), the 454 library preparation using a GS FLX Titanium General Library Preparation Kit, pyrosequencing, and transcriptomic assembly were all performed by the Zhongxin Biotechnology Company (Shanghai, China).

### Data analysis of the 454 sequencing data

The raw reads generated from the 454 sequencing were pre-processed to remove the adaptor primer and primer sequences, which were added during the construction of the cDNA library and the 454 sequencing, using SeqClean (version 86_64) and Newbler (version 2.5.3). Subsequently, low-complexity, low-quality, and very short (<50 bp) sequences were removed using Lucy (version 1.20p) (http://www.jcvi.org/cms/research/software/) and the trimmed sequences were assembled using Newbler (version 2.5.3) (http://my454.com/products/analysis-software/index.asp). The assembled isotigs and singlets in the ovary and testis libraries were merged and then clustered based on sequence similarity >95% using the CD-HIT software (version 4.0) (http://weizhong-lab.ucsd.edu/cd-hit/).

All the unique sequences that were obtained were compared against the NCBI non-redundant (Nr) protein sequence database using BLASTX with a cut-off E-value <1e-5 and protein identity >50%. Gene names were assigned based on the best BLAST hit among the matched sequences. Gene ontology analysis was conducted using the Blast2GO program [69, 70] and a GO analysis was employed to compare the annotated sequences based on their sequence similarity. GO terms from the biological process, molecular function, and cellular component GO categories were assigned to the annotated transcripts. In this paper, we have presented the results of the GO analysis based on level 2 terms, which describe general functional categories. The unique sequences were assigned to KEGG pathways using the online KEGG Automatic Annotation Server (KAAS) (http://www.genome.jp/kegg/kaas/) by searching the KEGG GENES database. The results, including KO (KEGG Orthology) assignments and automatically generated KEGG pathways, were output and the KEGG pathways were populated with the KO assignments.

### Identification of SSRs and SNPs

MISA software (http://pgrc.ipk-gatersleben.de/misa/) was used to detect microsatellites in all the isotigs and singlets. The search criteria were: di-nucleotide repeats ≥6, tri-nucleotide to hexa-nucleotide repeats ≥5, and the largest interval between two SSRs was ≤100 bases.

All the assembled reads in the isotigs were used as reference sequences to detect potential SNPs using ssahaSNP (http://www.sanger.ac.uk/resources/software/ssahasnp/). Based on the different bases at one position in the assembled sequences from the same isotig, SNP loci and insert/missing sites were predicted. Based on the the number of reads (n) at a specific SSR or SNP position, the number of SSRs and SNPs in an assembled sequence was calculated when n ≥1, n ≥3, and n ≥5.

### Identification of gonad-differentially and gonad-specifically expressed genes

BLASTN alignments were conducted to compare the unigene sequences of the testis and ovary pools. The best match (identity ≥90%) was taken as a pair of common gene and the rest of the matched sequences (identity <90%) were taken as gonad-specifically expressed sequences in the testis/ovary library. The Fisher *P* test was used to identify differentially expressed sequences between each pair of common genes. When the Fisher *P*-value <0.05, the two sequences representing the same gene in two samples were considered to be significantly differently expressed.

### qRT-PCR and semi-quantitative PCR analysis

We chose 60 genes with low *P*-values from the 4,021 gonad-differentially expressed genes for qRT-PCR verification. These 60 genes all exhibited large significant differences in expression between the ovaries and testes libraries. On the basis of the RPKM value, we also chose 68 ovary-specifically and 55 testis-specifically expressed genes with high expression levels (high RPKM values) from 10,522 ovary-specifically and 19,013 testis-specifically expressed genes libraries. The sequences of the 183 (60 + 68 + 55) differentially expressed genes and testis/ovary specifically expressed genes were chosen for design primers using Array Designer 4(http://premierbiosoft.com/dnamicroarray/index.html). The housekeeping gene 18S rRNA was used as an endogenous control.

qRT-PCR was performed for the 60 differentially expressed genes to verify whether their expression levels differed between the ovaries and testes. The PCR was performed in the Applied Biosystems 7500 real-time system (ABI 7500) using SYBR green PCR Master Mix as recommended by the manufacturer. Briefly, an aliquot of 3 μg of RNA pretreated with DNase I (37°C, 30 min) was used as a template for cDNA synthesis with random hexamers according to the user information for the Moloney murine leukemia virus (M-MLV) reverse transcriptase (Promega, Shanghai, China). PCR efficiency was determined for each primer pair by creating standard curves from serial dilutions to ensure that the E-value was more than 90% (Additional file 3: Table S4). The PCR was carried out at 95°C for 1 min, 40 cycles of 95°C for 15 s, 60°C for 1 min. Melting curves were plotted to make sure that a single PCR product was amplified for each pair of primers. The PCRs to detect all the target genes and 18S rRNA control gene were performed with three biological replicates and three technical replicates. The data for the three samples were used to calculate the mean of the relative quality (RQ) value, 2^-∆∆CT^ (∆CT = CT (cycle threshold) of target gene minus CT of 18S rRNA, ∆∆CT = ∆CT of any sample minus calibrator sample) and SE (Standard Error). Student's t-test was conducted using SPSS 20.0 (http://www-01.ibm.com/software/analytics/spss/). A significant difference was accepted at *P* <0.05 (two-tailed test). To ensure reliability of the results, PCR products were detected by gel electrophoresis using 1.5% agarose gel.

Semi-quantitative PCR was used first to detect the expression of testis/ovary specifically expressed genes. When a gene was found to be expressed in only in ovaries or testes, the gene was considered to be a testis/ovary-specifically expressed gene. For the genes that were expressed in both ovaries and testes, qRT-PCR was conducted to verify whether they exhibited expression differences between ovaries and testes. The original cDNAs were diluted 100-fold for the target genes and 40,000-fold for the 18S rRNA gene. The PCR cycle for both the specifically expressed genes and the 18S rRNA was as follows: 1 min at 95°C, then 40 cycles of 15 s at 95°C, 30 s at 60°C and 40 s at 72°C, followed by 10 min at 72°C. PCR products were detected by gel electrophoresis using 1.5% agarose gel. The primer sequences used for qRT-PCR and semi-quantitative PCR are listed in Table S5–S7 respectively.

### Ethics

The study has been approved by the legally ethical committee of Jimei University.

## Electronic supplementary material

Additional file 1: **Summaries of SSR (Table S1), SNP (Table S2), and important genes (Table S3) in the transcriptome of** ***S. paramamosain.*** (ZIP 182 KB)

Additional file 2: **Electrophoretic result (Figure S1) and expression pattern (Figure S2) for** ***Sp-VgR*** **from green mud crab tissues in different stages of developing ovaries by qRT-PCR.** (ZIP 45 KB)

Additional file 3: **Amplification efficiency of qRT-PCR (Table S4) and confirmation of gonad-differentially (Table S5), testis-specifically (Tables S6), and ovary-specifically (Table S7) expressed transcripts of** ***S. paramamosain*** **by qRT-PCR.** (ZIP 45 KB)
