# Supplementary material for: Transcriptome analysis of the differences in gene expression between testis and ovary in green mud crab (Scylla paramamosain)
Source: BMC Genomics. 2014 Jul 11;15(1):585. doi: 10.1186/1471-2164-15-585 (PMC4124137; doi:10.1186/1471-2164-15-585)
Supplement: Supplementary file 1 — Additional file 1: Summaries of SSR (Table S1), SNP (Table S2), and important genes (Table S3) in the transcriptome of S. paramamosain. (ZIP 182 KB) [file 12864_2013_6318_MOESM1_ESM.zip › 9622841049436572_fig12.pdf]

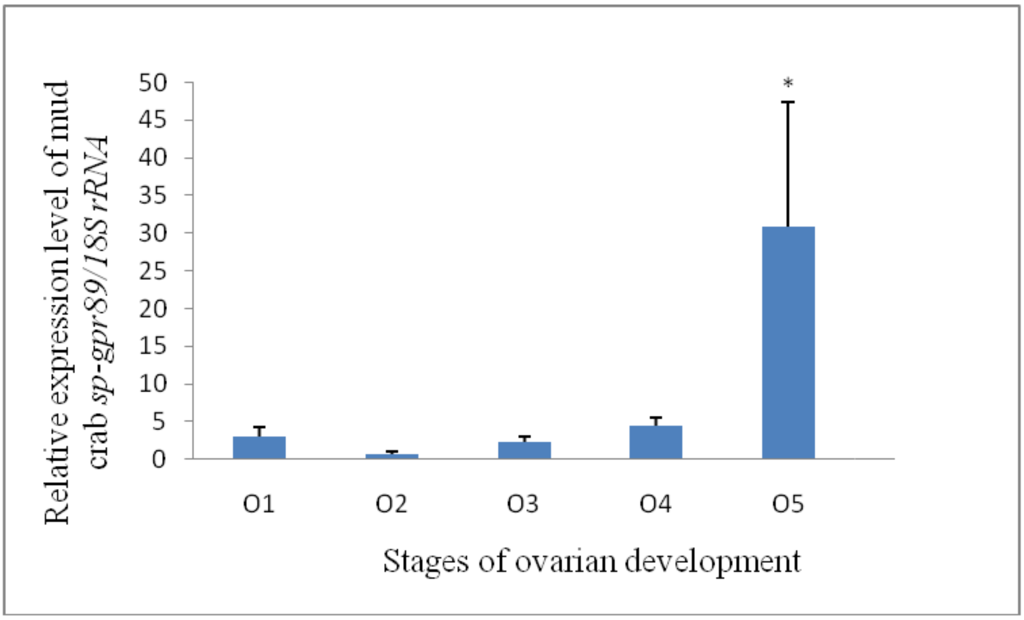

Fig. S2 Expression pattern of *Sp-gpr*89 in different stages of developing ovaries by qRT-PCR

Data are presented as means ± SEM of at least three separate individuals. * indicates significantly differential expression ($p < 0.05$).
